# Light-intensity grazing improves alpine meadow productivity and adaption to climate change on the Tibetan Plateau

**DOI:** 10.1038/srep15949

**Published:** 2015-10-30

**Authors:** Tao Zhang, Yangjian Zhang, Mingjie Xu, Juntao Zhu, Michael C. Wimberly, Guirui Yu, Shuli Niu, Yi Xi, Xianzhou Zhang, Jingsheng Wang

**Affiliations:** 1 Key Laboratory of Ecosystem Network Observation and Modeling, Institute of Geographic Sciences and Natural Resources Research, Chinese Academy of Sciences, Beijing 100101, China; 2 College of agronomy, Shenyang Agricultural University, Shenyang 110866, China; 3 Center for Excellence in Tibetan Plateau Earth Sciences, Chinese Academy of Sciences, Beijing 100101, China; 4 University of Chinese Academy of Sciences, Beijing 100049, China;; 5 Geospatial Sciences Center of Excellence, South Dakota State University, Brookings, SD 57007, United States

## Abstract

To explore grazing effects on carbon fluxes in alpine meadow ecosystems, we used a paired eddy-covariance (EC) system to measure carbon fluxes in adjacent fenced (FM) and grazed (GM) meadows on the Tibetan plateau. Gross primary productivity (GPP) and ecosystem respiration (Re) were greater at GM than FM for the first two years of fencing. In the third year, the productivity at FM increased to a level similar to the GM site. The higher productivity at GM was mainly caused by its higher photosynthetic capacity. Grazing exclusion did not increase carbon sequestration capacity for this alpine grassland system. The higher optimal photosynthetic temperature and the weakened ecosystem response to climatic factors at GM may help to facilitate the adaption of alpine meadow ecosystems to changing climate.

Land use and associated management practices can affect ecosystem nutrient, carbon and water cycling by acting on plant photosynthesis, respiration and energy partitioning^1^,^2^,^3^. Grazing is the most ubiquitous land use practice for managed grassland ecosystems^4^,^5^.It exerts strong influences on ecosystem carbon budget, energy balance, and ecosystem function^6^,^7^. Knowledge of the responses of carbon fluxes to grazing is essential for developing sustainable management plans, coping with consequences of climate change, and mitigating related effects^8^,^9^.

Alpine meadows comprise the representative vegetation on the plateau and cover about 35% of its total area^10^. After thousand-years of years of grazing in these meadows, the practice has become an indispensable agriculture activity for the Tibetan grassland in terms of both regional sustainable development and culture. Over time, the Tibetan grassland has adapted to the effects of both natural climatic variations and anthropogenic disturbances caused by grazing^11^. In past decades, however, overgrazing has resulted in environmental deterioration and even desertification in the alpine grassland ecosystem, consequently generating considerable quantities of carbon efflux^10^,^12^.

It is still far from clear how grazing acts on biogeochemical cycling of the Tibetan grassland, and whether grazing depresses or stimulates grassland productivity. Grazing effects vary with stocking densities and grazing period length^3^. In general, grazing leads to decreased leaf area, in turn lowered ecosystem carbon acquisition. However, older leaves in ungrazed grasslands may possess lower ecosystem carbon fixation ability^12^,^13^. Grazing also substantially modifies canopy structure and land surface energy balance, altering microclimates in such aspects as soil temperature and moisture and potentially facilitating more rapid plant growth^14^,^15^. In contrast, slowed nitrogen dynamics under long-term grazing can negatively influence plant growth and soil microbial activities^16^. All these changes have the potential to alter the magnitude of ecosystem carbon uptake and emission^4^,^17^.

The Tibetan Plateau ecosystem is a relatively fragile system and is sensitive to climate changes^18^. It acts as a critical “first response region” to climate change in China and East Asia^19^. Climatic factors and grassland management both have strong influences on the seasonal and inter-annual dynamics of carbon fluxes^3^,^7^.Grazing mediates the relationships between ecosystem function and carbon flux variability by means of altered canopy structure and plant physiology^4^. Changes in ecosystem function can dominate the inter-annual variability of carbon fluxes^20^ and determine the ecosystem adaptability to climate change^4^. Thus, quantifying grazing effects on carbon dioxide uptake and emissions of alpine grassland systems is critical for understanding their role in global climate change.

To effectively monitor grazing effects on carbon flux, the eddy covariance technique is a reliable source of *in situ* measurements with its capability of automatic and high-frequency measurements of gas and energy fluxes. There have been few prior studies that have used the eddy covariance technique on paired treatments of grazing-nongrazing^12^,^21^, especially for actively managed grasslands^22^,^23^. These research gaps constrain our capacity to adequately address grazing disturbance effects. In particular, knowledge about grazing impacts on carbon fluxes is necessary to scale up carbon budget from measured sites to larger terrestrial entities^24^. To this end, in this study we focused on carbon fluxes of alpine meadow ecosystem under grazed and ungrazed treatments in the same environmental setting to examine how carbon fluxes respond to grazing in alpine meadows on the Tibetan Plateau. Our main research questions were: 1) Does light-intensity grazing could maintain higher ecosystem productivity compared with short-term fencing in these alpine meadows? and 2) How does grazing affect the responses of carbon fluxes to climatic variations?

## Data and Methods

### Site description

Our study site is located in the core zone of the Northern Tibetan Plateau, where a typical alpine *Kobresia pygmaea* meadow is distributed. The region belongs to the plateau subfrigid monsoon climate, with no absolute frost-free period throughout the year. The soil freezing period is from October to next May, and the annual mean air temperature is −1.9 °C. The mean annual rainfall is 380 mm, with 80% falling between June and September. The annual average sunlight exceeds 2886 h. The soil is classified as meadow soil with sandy loam. The vegetation is dominated by *Kobresia pygmaea*, accompanied by *Potentilla bifurca, Potentilla saundersiana, Leontopodium pusillum, and Carex moorcroftii*.

**Figure 1:**
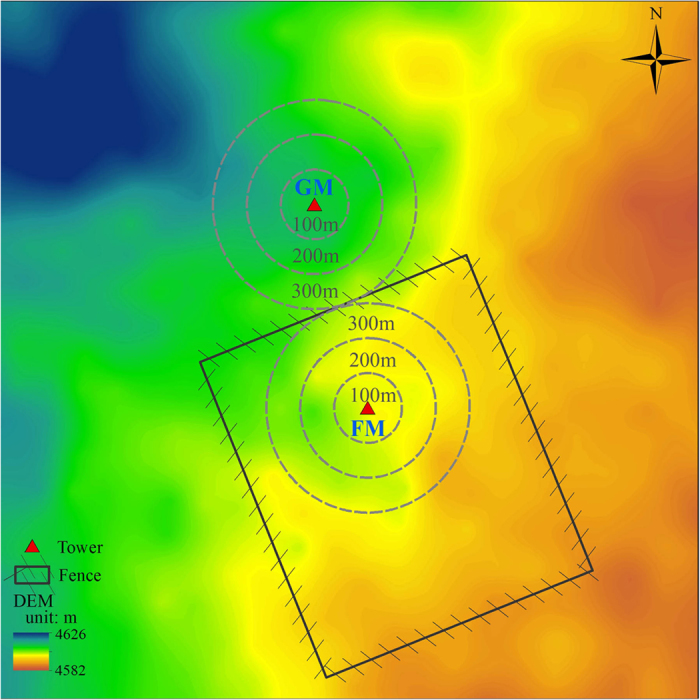
Area relief map of the study site based on a Digital Elevation Model (DEM) with 100 m equidistance lines. The paired towers are located in the fenced and grazed meadows, respectively, with 600 m distance between two towers.

The grazing exclusion treatment meadow (FM, 31.643686°N, 92.009683°E, 4598 m a.s.l.) has been fenced since October 2011 and lies in the same watershed with the grazed treatment meadow (GM, 31.648722°N, 92.007208°E, 4607 m a.s.l.), which ensures that the two treatment sites experience similar local climates. The topographic map around the paired eddy covariance towers is shown in Fig. 1. Prior to the grazing exclusion treatment, vegetation and climates were quite similar between fenced and grazed sites. They exhibited no significant net ecosystem exchange (NEE) differences (P = 0.997) and had relatively homogeneous vegetation, dominated by *Kobresia pygmaea* (>65%). Before the experiment was established, both GM and FM was grazed using a common local grazing intensity of <0.5 sheep and <1.2 yak unit ha^−1^ half-y^−1^ for over 20 years, and the same grazing intensity maintained during the experiment at GM. Under the atmospheric neutral stability assumption, the footprint analysis^25^ showed that approximately 95% and 97% of the measured scalar fluxes originated from the FM and GM towers, respectively.

### Experimental measurements

The open-path eddy covariance (OPEC) system was used to measure carbon and water vapor fluxes at 2.3 m above the ground. The OPEC system consists of a 3-D sonic anemometer (Model CSAT-3, Campbell Scientific Inc., Logan, UT, USA) to measure three-dimensional wind speed and temperature fluctuations, and an infrared gas analyzer (Model LI-7500A, Li-cor Inc., Lincoln, NE, USA) to measure CO_2_ and water vapor densities. All signals were sampled at a frequency of 10 Hz. The CO_2_ and H_2_O fluxes were calculated and recorded at 30 min intervals by a CR3000 datalogger (Model CR3000, Campbell Scientific).

The meteorological conditions, including solar radiation, net radiation (Rn) and photosynthetically active radiation (PAR), were observed at 1.5 m height above the ground using a four-component net radiometer (Model CNR-1, Kipp&Zonen, Netherlands) and a quantum sensor (LI190SB, Li-cor Inc.). Air temperature (Ta) and relative humidity (RH) were measured at 1.8 m height (Model HMP45C, Vaisala Inc., Helsinki, Finland). Soil temperatures (Ts) and soil water contents (SWC) were recorded at four depths (0.05, 0.1, 0.2 and 0.5 m) with thermometers (109-L, Campbell Scientific) and TDR probes (Model CS616-L, Campbell Scientific), respectively. The above meteorological data were summarized to half-hour intervals, except precipitation (PPT), which was measured continuously by the tipping bucket rain gauge (TE525MM-L, Campbell Scientific). All meteorological data were recorded using a CR1000 datalogger (Model CR1000, Campbell Scientific).

Replicate samples (n = 10) for aboveground biomass were collected during June—August from 2012 to 2014 once a month by clipping vegetation of 0.5 × 0.5 m^2^ quadrats within a radius of 250 m around each observation tower.

### Data processing

The analyses were based on half-hour means of CO_2_ fluxes from 2012 to 2014. The flux data were first aligned with the coordinate system of mean wind direction using the three-dimensional rotation^26^,^27^, then corrected for bias caused by air density variation due to heat and water vapor fluctuations using the Webb, Pearman and Leuning density correction (WPL), a correction for the effects of air density fluctuations^28^. Anomalous or spurious values caused by interference from rain, extreme cloud cover, dew, hoarfrost, or birds were excluded from the analysis.

The CO_2_ flux measured by the eddy covariance technique represents the net ecosystem CO_2_ exchange (NEE), which is equivalent to the net ecosystem productivity (NEP) multiplied by −1. The NEE was partitioned into gross primary productivity (GPP) and ecosystem respiration (Re)^29^. The daytime NEE was assumed to be valid for our analysis when photosynthetically active radiation (PAR) was greater than 1 μmol m^−2^ s^−1^. In calculating nighttime NEE, PAR was always less than 1 μmol m^−2^ s^−1^.To avoid possible flux underestimation under stable conditions during the night, effects of friction velocity u* were examined statistically. This analysis determined that the threshold values of u* were 0.16 m s^−1^ for fenced and 0.17 m s^−1^ for grazed meadows in 2012, 0.11 m s^−1^ for fenced and 0.13 m s^−1^ for grazed meadows in 2013 and 0.11 m s^−1^ for fenced and 0.14 m s^−1^ for grazed meadows in 2014, respectively. The negative nighttime NEE were excluded from our analysis. All the missing data for each 30 min were filled using the methods of linear fitting, nonlinear fitting and mean diurnal variations^27^,^30^. The number of missing observations, filtered values and the total observations were counted (Table 1). The energy balance closure was evaluated based on half-hourly observations using the energy balance ratio (EBR).


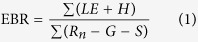


Where LE represents the latent heat flux, H represents sensible heat flux, Rn represents net radiation, G represents soil heat flux, and S represents canopy heat storage. The closer EBR is to 1, the better the energy balance closure.

To investigate grazing affects, we used data from 2012 to 2013 when the grazing effect was strongest (The differences between FM and GM were significant, P < 0.01). We used the Michaelis–Menten equation^31^ to calculate the ecosystem light response:


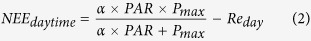


where the unit of NEE_daytime_ and PAR are mg CO_2_ m^−2^ s^−1^ and μmol m^−2^ s^−1^, respectively; α (mgCO_2_ μmolPhoton^−1^) is the apparent quantum yield or the initial slope of the light response curve; P_max_ (mgCO_2_ m^−2^ s^−1^) is the maximum apparent photosynthetic capacity of the canopy; and Re_day_ (mgCO_2_ m^−2^ s^−1^) is the ecosystem respiration during daytime.

### Statistical analysis

To determine the driving factors on carbon fluxes, stepwise multiple linear regression analysis was employed. First, we conducted a comprehensive analysis that used Grazing (value = 0 at FM; value = 1 at GM), Rn, Ta, Ts, SWC and VPD as independent variables and NEP, Re and GPP as separate dependent variable. Second, to explore the relative contribution of each factor to carbon fluxes under the grazing or non-grazing treatment, we conducted partial correlation analysis of carbon fluxes (NEP, Re and GPP) against climatic factors (Rn, Ta, Ts, SWC and VPD) for FM and GM sites separately. By comparing the two response patterns (with and without grazing being included), we can simultaneously obtain knowledge about how grazing affects carbon flux response to climate change.

The one-way analysis of variance (ANOVA) was used to examine differences of carbon fluxes and environmental variables between the fenced and grazed sites. We used OriginPro 8 SR0 software to perform all the statistical analysis and the level of significance was set at 0.05.

## Results

### Characteristics of environmental variables and biotic environment

**Figure 2:**
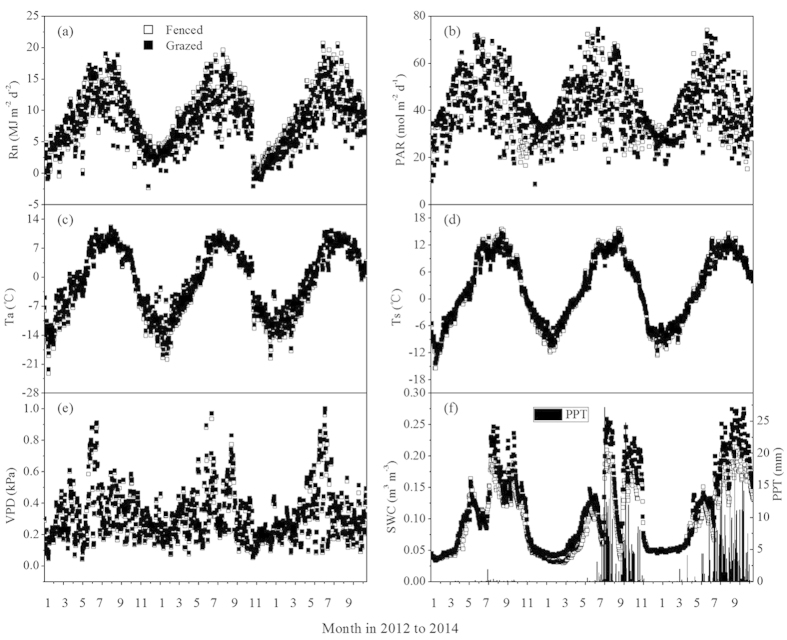
Monthly change in daily net radiation (Rn, (a)), photosynthetically active radiation (PAR, (b)), air temperature (Ta, (c)), soil temperature (Ts, (d)) at 5 cm depth, vapor pressure deficit (VPD, (e)), and soil volumetric water content (SWC, f) at 5 cm depth for the fenced and grazed alpine meadows, 2012–2014. Precipitation (PPT) data were available since June 2013.

The net radiation (Rn), photosynthetically active radiation (PAR), air temperature (Ta), soil temperature (Ts), and vapor pressure deficit (VPD) all exhibited unimodal dynamics at both FM and GM within a year. The annual total radiations were similar between FM and GM (Fig. 2(a,b)). The average daily Ta was higher at GM than that at FM (Fig. 2(c), P = 0.048). The daily mean Ts was higher at FM than that at GM in growing season (P = 0.024), while the daily mean Ts was higher at GM in non-growing season (Fig. 2(d), P < 0.01). Both Ta and Ts reached their peaks from early July through late August at the two sites. The VPD was always lower at FM than that at GM across the three years (Fig. 2(e), P < 0.01). The mean SWC was lower at FM than at GM (P < 0.01) and the seasonal variations of SWC showed strong correspondence to rain events throughout each growing season (Fig. 2(f), P < 0.01). A severe drought in August 2013 resulted in a continuous decrease of SWC from early August until September (Fig. 2(f)) and a VPD peak in August (Fig. 2(e)).

**Figure 3:**
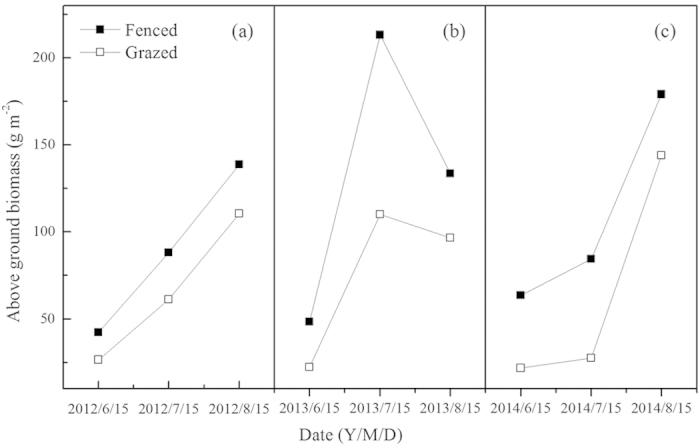
Variations of aboveground biomass in peak season for the fenced and grazed alpine meadows for year (a) 2012, (b) 2013, and (c) 2014.

Both fenced and grazed grasslands achieved their maximum aboveground biomass in August 2012 and 2014 (Fig. 3(a,c)). The maximum aboveground biomass was 139 and 110 g m^−2^ for FM and GM in 2012, and 188 and 160 g m^−2^ in 2014, respectively. In 2013, an earlier precipitation peak arrived in July and resulted in an earlier biomass peak (Fig. 2(f)), with a maximum biomass values of 213 and 110 g m^−2^ for FM and GM, respectively. The biomass decreased sharply in August due to evident water stress in the alpine meadows.

### Daily and monthly dynamics of carbon fluxes

**Figure 4:**
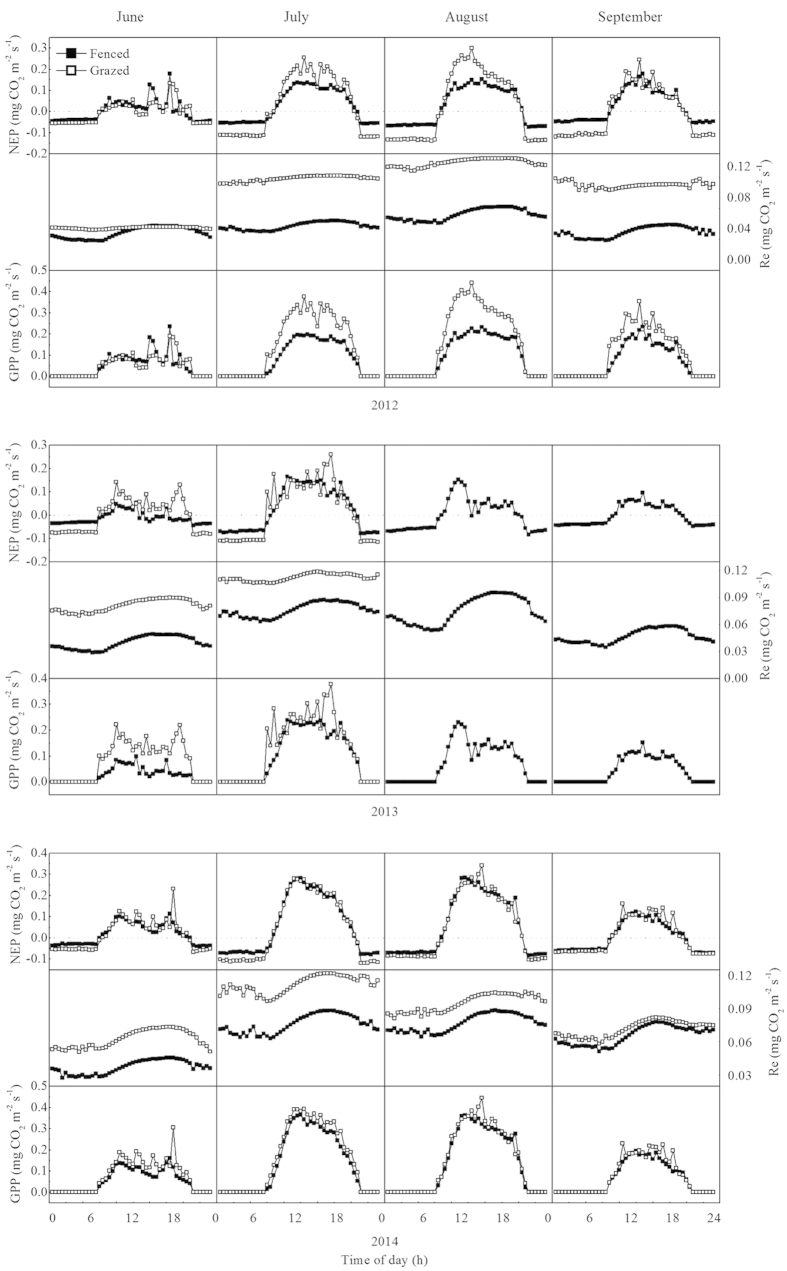
Daily variations of the monthly means of carbon fluxes (net ecosystem productivity (NEP), ecosystem respiration (Re), and gross primary productivity (GPP)) at the fenced and grazed meadows from June to September. The 30 min data shown are means for all days in each month for the two ecosystems. Flux data were absent at the grazed meadow in August and September 2013.

The daily dynamics of carbon fluxes (NEP, Re, and GPP) varied substantially between FM and GM within the growing seasons from 2012 to 2014 (Fig. 4). The GM had higher carbon flux peak values than those of FM, but their differences decreased throughout the experiment. The largest daily carbon fluxes in the two treatments were recorded for August in 2012 and 2014, and July in 2013. The NEP values were 0.152 and 0.299 mgCO_2_ m^−2^ s^−1^ for FM and GM in 2012, 0.285 and 0.342 mgCO_2_ m^−2^ s^−1^for FM and GM in 2014, and 0.166 and 0.260 mgCO_2_ m^−2^ s^−1^ for FM and GM in 2013. These peaks occurred simultaneously with the maximum biomass (Fig. 3). The NEP and GPP were greater before noon than in the afternoon, especially in August 2013—a severe drought month, leading to an asymmetrical distribution of NEP and GPP around noon. The daily maximum of NEP and GPP occurred at 11:00, with values of 0.152 and 0.229 mgCO_2_ m^−2^ s^−1^, respectively. The daily maximum Re occurred around 16:00–17:00, and the values were higher at GM than at FM throughout each growing season. Both ecosystems showed net carbon gains in July and August and neutral values in June and September. However, the net carbon sink turned to a weak carbon source in August 2013, with a NEP value of −1.64 gC m^−2^ in that month.

**Figure 5:**
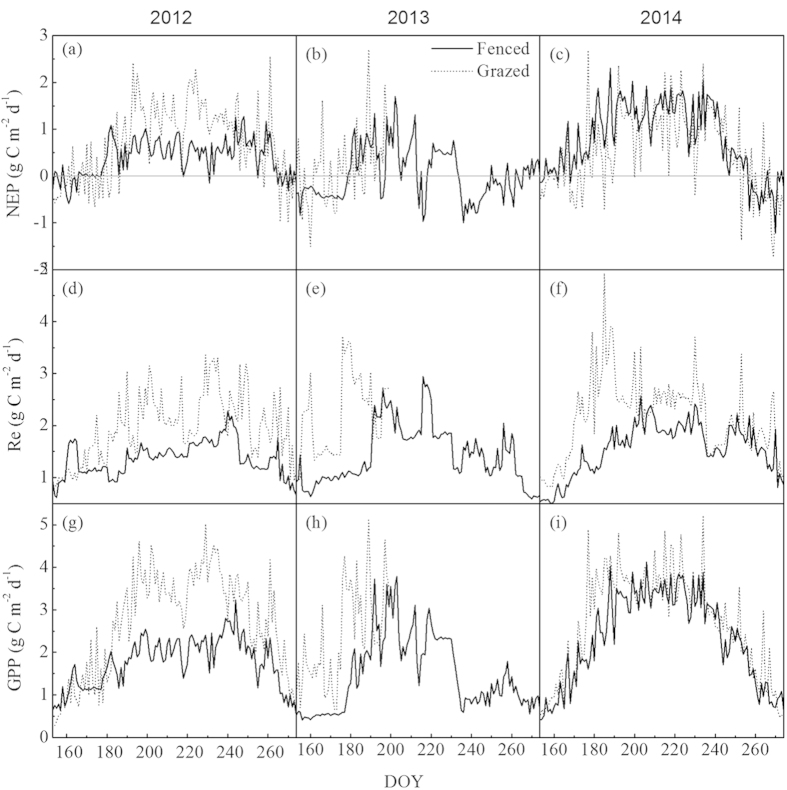
Daily variations of NEP, Re, and GPP for the fenced and grazed meadows during the growing season from 2012 to 2014.

During the growing seasons from 2012 to 2014 (Fig. 5), the means of NEP were 0.39, 0.04, 0.79 gC m^−2^ d^−1^ for FM and 0.72, 0.19, 0.55 gC m^−2^ d^−1^ for GM, respectively. The means of Re were 1.36, 1.43, 1.55 gC m^−2^ d^−1^ for FM and 1.96, 2.19, 2.16 gC m^−2^ d^−1^ for GM, respectively. The means of GPP were 1.75, 1.47, 2.34 gC m^−2^ d^−1^ for FM and 2.68, 2.38, 2.71 gC m^−2^ d^−1^ for GM, respectively. One thing to note is that the carbon flux means at GM in 2013 were calculated using the data from DOY 153 to 200. Generally, carbon fluxes at GM were greater than those at FM in 2012. The NEP and GPP rebounded rapidly in the following two years, while recoveries of Re were not obvious. The carbon flux differences between FM and GM were obvious in 2012 and 2013 (P < 0.01), which provided an opportunity to investigate grazing effects on this alpine ecosystem.

### Comparison of photosynthetic capacity between FM and GM

**Figure 6:**
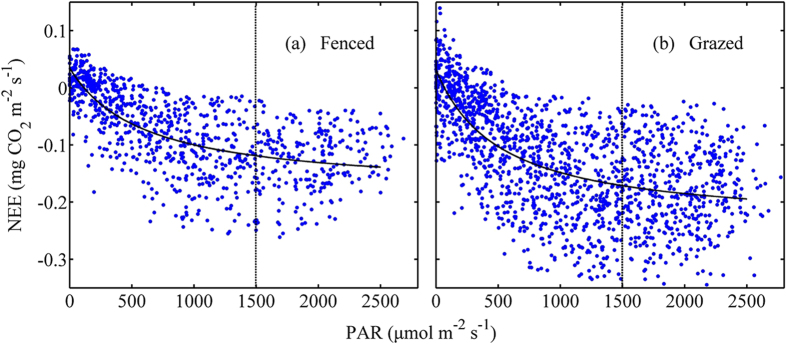
Relationship between net ecosystem CO_2_ exchange (NEE) and light intensity at the fenced and grazed meadows during DOY 180–250, 2012–2013. The curves are fitted results using Eq. (1) based on the observed data (n_Fenced_ = 902, n_Grazed_ = 1565). The observed NEE were separated into two sections with a threshold of 1500 μmol m^−2^ s^−1^.

Solar radiations were very strong at both sites (Fig. 2(a,b)). The NEE increased with light intensity and reached its maximum around 1500 μmol m^−2^ s^−1^ of PAR, then leveled off (Fig. 6). The α was 0.000358 mgCO_2_μmolPhoton^−1^ for the FM grassland and 0.000531 mgCO_2_μmolPhoton^−1^ for the GM grassland, respectively. The P_max_ was 0.21 mgCO_2_ m^−2^ s^−1^ and 0.27 mgCO_2_ m^−2^ s^−1^ for the FM and GM grasslands, respectively.

Under any environment group (Table 2), α and P_max_ at the GM grasslands were greater than those at FM, indicating a greater carbon uptake capacity by the prior one. The α increased, but P_max_ followed a flat trend along rising Ta at FM. At GM, both α and P_max_ increased with rising Ta. The optimum temperatures were 10–13 °C and ~13 °C at FM and GM, respectively.

The value of α increased with enhanced SWC at both FM and GM. Across the two treatment grasslands, P_max_ was greatest when SWC was in the range between 0.13 and 0.18 m^3^ m^−3^. The maximum α in GM and FM occurred in the VPD range between 0.3 and 0.6 and between 0.6 and 0.9, respectively, with a greater value for the prior treatment than for the latter one. Both ecosystems achieved their maximum P_max_ when VPD fell in the range between 0.6 and 0.9 kPa, and P_max_ of GM was 29% greater than that of FM.

### Drivers of carbon fluxes

We analyzed the relationships between carbon fluxes (i.e., NEP, Re and GPP) and their potential drivers (Grazed, Rn, Ta, Ts, SWC and VPD) using multiple linear stepwise regression (Table 3). In this alpine ecosystem, grazing had the strongest influence on Re, and the Rn had the strongest influence on NEP and GPP. Grazing was the second most important driver of GPP.

The partial correlation analysis of carbon fluxes against climatic factors was conducted for the fenced and grazed meadow separately, to differentiate the role of grazing from climatic factors (Table 4). Rn was the main driver on NEP and GPP in both ecosystems. Re was mainly controlled by Ts at FM and by SWC at GM. In general, grazing weakened the correlations of GPP and NEP with climatic factors. Also, grazing weakened the correlations of Re with Rn, Ta and Ts, but enhanced the correlation of Re with SWC and VPD.

## Discussions

### Response of carbon budget to grazing in the alpine meadow

The daily amplitudes of NEP at FM are within the range observed for grassland ecosystems with similar low leaf area index on the Tibetan Plateau^32^,^33^. A wider range of temporal variation has been identified at GM than at FM, though it has been fenced only for three years. This result indicates more carbon uptake in daytime and more carbon emission in nighttime at GM than at FM, which is in accord with similar studies conducted in tallgrass prairies in US^12^ and desert steppe on the Mongolian Plateau^21^. The wider daily NEP amplitude at GM during growing seasons emphasizes the importance of including anthropogenic activities disturbances in future carbon flux modeling work.

The grazed meadow acted as a stronger carbon sink than the fenced meadow in the first two years of fencing. This result shows that lightly-grazed alpine meadows can contribute more to atmospheric carbon uptake than similar meadows where grazing is excluded^12^,^21^. Similar findings have been reported in mixed prairie in Nevada, USA^34^,^35^. The higher GPP and Re at GM than that at FM were also in accord with previous studies^36^ conducted for shortgrass steppe in northeastern Colorado, US.

The short-term fencing decreased ecosystem productivity. However, the ecosystem productivity of FM increased over time and reached a level almost equal to the GM site in the third year of fencing. Possible reasons might be that the ecosystem had adapted to the new environment^37^, and that grazing intensity in this alpine ecosystem is light. This experiment provided a way to judge the grazing intensity of a grazed grassland ecosystem. It also shows that in overgrazed areas, fencing would be a good method to facilitate ecosystem recovery.

### Response of photosynthetic capacity to grazing in various environmental conditions

Ecosystem gross photosynthesis is driven by biotic and abiotic factors, as well as their interactions^30^,^30^,^38^. The higher ecosystem carbon sequestration potential at GM than at FM might be caused by a temporary negative response of the latter ecosystem to non-grazing. More suitable microclimate at GM might be another reason^21^. Temperature is a critical environmental factor for plant growth on the Tibetan Plateau^30^. The alpine plants have been acclimated to the long-term low temperature conditions^39^ by developing photosynthetic and other related organisms optimal for low temperatures^30^. The higher optimum temperature for carbon uptake at GM than that at FM indicates that light-intensity grazing might improve alpine meadow ecosystem productivity under global warming.

Carbon assimilation was significantly reduced in both ecosystems when Ta < 7 °C. Both α and P_max_ increased with Ta at GM, while P_max_almost leveled off along increased temperature at FM. This is the direct result of greater carbon uptake at GM than FM during the active growing season in the first two years of fencing. Grazing also indirectly affected carbon uptake by changing Ts^4^. Reduced biomass of litter and dead standing plants caused by grazing can weaken the greenhouse effect of canopy, and maintain suitable temperature and light conditions for plant growth.

Generally, water deficiency causes decreased net carbon uptake and internal leaf CO_2_ concentrations through adjusting leaf stomata^40^. In this study, α increased with SWC in both treatments, which was consistent with other studies^41^,^42^. The GM had higher SWC than FM due to reduced evapotranspiration caused by grazing^13^. Therefore, the GM had a greater potential for carbon sequestration. The maximum P_max_ appeared when SWC fell in the rang within 0.13–0.18, not at the point of maximum SWC, which indicates that moisture was not a limiting factor in this alpine meadow. The higher α and P_max_ for GM than for FM might be related to the grazed meadow’s more physiologically active leaves and higher single-leaf photosynthetic capacity^21^ under the situation of reduced green net primary productivity (GNPP) and leaf area index (LAI) caused by grazing^14^.

Normally, there is a reduction in carbon uptake at higher VPD because of stomatal closure under drought conditions^43^. In this study, the lowest carbon uptake was not observed when VPD was greater than 0.9. On the contrary, both ecosystems had lower carbon sequestration under when VPD was lower than 0.3 because of low temperature. This indicated that Ta, not VPD was the major factor regulating ecosystem carbon uptake of this alpine meadow during active growing seasons.

### Grazing effects on the alpine meadow ecosystem productivity

Grazing can increase ecosystem photosynthesis and respiration. Light-intensity grazing will stimulate development of new leaves, whose higher photosynthetic capacity can compensate for reduced photosynthesis caused by thinned leaves^12^. In addition, grazing can improve soil fertility by increasing available nitrogen, which generates another avenue for enhancing ecosystem productivity^44^ and ecosystem respiration. The reason is that respiration is limited by supplies of carbohydrates fixed through photosynthesis^30^. Animal waste decomposition releases a large amount of CO_2_, which exerts a significant influence on ecosystem respiration. The improved microclimates at GM^45^,^46^ and influences of animal saliva on grass growth^47^ may also be responsible for increased carbon fluxes. For example, an open canopy allows for more adequate sunshine at GM, consequently prolonging carbon uptake time during thedaytime^21^. In contrast, the denser shading at FM can reduce carbon fluxes^48^,^49^.

The stepwise multiple linear regression analysis revealed the importance of grazing in controlling the carbon budget of this alpine meadow within three years since fencing. The absolute response magnitude of carbon fluxes to climate change should increase with plant biomass^4^. By reducing aboveground biomass, grazing lessened the strength of flux—climate correlations. It means that grazing weakened the response of carbon fluxes to climatic factors^3^,^4^, and light-intensity grazing might thus improve adaptation of this alpine ecosystem to changed climates^50^.

## Additional Information

**How to cite this article**: Zhang, T. *et al.* Light-intensity grazing improves alpine meadow productivity and adaption to climate change on the Tibetan Plateau. *Sci. Rep.* **5**, 15949; doi: 10.1038/srep15949 (2015).

## Figures and Tables

**Table 1:** Number of half-hourly observations obtained from eddy flux measurements.

	Fenced				Grazed			
	missing data	filtered data	total data	EBR	missing data	filtered data	total data	EBR
2012	7597	6356	17568	0.85	8517	5187	17568	0.86
2013	7182	6351	17520	0.83	8117	6327	17520	0.77
2014	5483	6666	17520	0.91	4137	7131	17520	0.89

*EBR is energy balance ratio.

**Table 2:** Estimated coefficients describing the rectangular hyperbolic responses of daytime NEE to incident PAR (equation (2)) and corresponding average meteorological conditions under different air temperature (Ta), soil water content (SWC), and vapor pressure deficit (VPD) levels during the vegetation active periods of DOY 180–250 in the fenced meadow (FM) and grazed meadow (GM) from 2012 to 2013.

		α	P_max_	Re_day_	Ta	VPD	Ts	SWC	R^2^	P	data volume
	(mgCO_2_ μmolPhoton^−1^)		(mgCO_2_ m^−2^ s^−1^)		(°C)	(kPa)	(°C)	(m^3^ m^−3^)			
Ta < 7	Fenced	0.000195	0.23	0.019	5.79	0.22	6.25	0.16	0.66	<0.01	215
	Grazed	0.000326	0.25	0.011	5.62	0.20	7.13	0.18	0.58	<0.01	250
7 ≤ Ta < 10	Fenced	0.000380	0.25	0.034	8.50	0.40	10.62	0.16	0.42	<0.01	214
	Grazed	0.000804	0.29	0.044	8.30	0.34	9.18	0.19	0.41	<0.01	384
10 ≤ Ta < 13	Fenced	0.000667	0.24	0.074	11.48	0.55	13.95	0.16	0.49	<0.01	268
	Grazed	0.000941	0.35	0.099	11.26	0.51	11.46	0.19	0.41	<0.01	479
Ta ≥ 13	Fenced	0.000629	0.23	0.084	14.50	0.84	17.12	0.17	0.40	<0.01	205
	Grazed	0.000947	0.34	0.125	14.59	0.85	15.27	0.19	0.39	<0.01	457
SWC < 0.13	Fenced	0.000278	0.22	0.036	8.66	0.46	12.28	0.11	0.40	<0.01	168
	Grazed	0.000444	0.24	0.021	9.19	0.36	10.13	0.12	0.33	<0.01	82
0.13 ≤ SWC < 0.18	Fenced	0.000350	0.23	0.036	9.46	0.54	11.93	0.16	0.51	<0.01	478
	Grazed	0.000482	0.31	0.037	10.05	0.56	11.68	0.16	0.46	<0.01	602
0.18 ≤ SWC < 0.23	Fenced	0.000500	0.20	0.034	10.88	0.44	12.30	0.19	0.53	<0.01	256
	Grazed	0.000557	0.27	0.035	10.86	0.53	11.27	0.21	0.44	<0.01	586
SWC ≥ 0.23	Fenced	—	—	—	—	—	—	—	—	—	—
	Grazed	0.000748	0.22	0.024	11.04	0.45	11.02	0.24	0.40	<0.01	295
VPD < 0.3	Fenced	0.000277	0.26	0.023	5.57	0.17	7.62	0.16	0.53	<0.01	270
	Grazed	0.000484	0.29	0.017	6.52	0.18	8.24	0.19	0.45	<0.01	425
0.3 ≤ VPD < 0.6	Fenced	0.000574	0.25	0.065	10.19	0.44	11.63	0.16	0.42	<0.01	316
	Grazed	0.001204	0.36	0.115	10.47	0.45	10.49	0.20	0.38	<0.01	578
0.6 ≤ VPD < 0.9	Fenced	0.000800	0.27	0.104	12.12	0.75	15.42	0.16	0.46	<0.01	222
	Grazed	0.001054	0.38	0.144	12.54	0.72	13.23	0.19	0.41	<0.01	357
VPD ≥ 0.9	Fenced	0.000485	0.23	0.079	14.32	1.03	18.73	0.15	0.54	<0.01	94
	Grazed	0.000777	0.33	0.113	15.26	1.05	16.73	0.18	0.40	<0.01	205

Eq. (2) was used to fit the regression coefficients based on the observed data.

**Table 3:** The results of multiple linear stepwise regression analysis of carbon fluxes (NEP, Re and GPP) against the potential drivers (Grazed, Rn, Ta, Ts, SWC and VPD) during the active vegetation period from 2012 to 2014.

regression equation	F	P	Grazed	Rn	Ta	Ts	SWC	VPD
NEP = 0.038–0.01Grazed + 0.0003Rn + 0.008Ta–0.005Ts + 0.23SWC–0.081VPD	624.1	<0.01	−0.05**	0.53**	0.14**	−0.11**	0.09**	−0.12**
Re = 0.104 + 0.051Grazed + 0.000006Rn + 0.001Ta–0.0004Ts–0.26SWC + 0.012VPD	707.8	<0.01	0.63**	0.05**	0.03*	0.03*	−0.30**	0.06**
GPP = 0.138 + 0.04Grazed + 0.0003Rn + 0.008Ta–0.004Ts–0.067VPD	789.2	<0.01	0.21**	0.52**	0.14**	−0.10**	—	−0.10**

^*^indicates that the correlation is significant at the 0.05 level;

^**^indicates that the correlation is significant at the 0.01 level

The Grazed, Rn, Ta, Ts, SWC and VPD columns represent the partial correlation coefficients between carbon fluxes and each climatic factor, respectively.

**Table 4:** The partial correlation coefficients between carbon fluxes and climatic factors during the active vegetation periods from 2012 to 2014 at FM (Fenced)and GM (Grazed).

regression equation	F	P	Rn	Ta	Ts	SWC	VPD
Fenced: NEP = −0.009 + 0.0003Rn + 0.011Ta–0.007Ts + 0.46SWC–0.085VPD	350.2	<0.01	0.538**	0.205**	−0.153**	0.134**	−0.128**
Grazed: NEP = 0.056 + 0.0003Rn + 0.004Ta–0.004Ts + 0.155SWC–0.061VPD	411.2	<0.01	0.511**	0.076**	−0.079**	0.067**	−0.093**
Fenced: Re = 0.051 + 0.00001Rn + 0.001Ta + 0.002Ts–0.053SWC–0.006VPD	272.6	<0.01	0.112**	0.061**	0.299**	−0.086**	−0.049*
Grazed: Re = 0.191–0.002Ts–0.329SWC + 0.034VPD	245.4	<0.01	—	—	−0.141**	−0.375**	0.201**
Fenced: GPP = 0.043 + 0.0003Rn + 0.012Ta–0.004Ts + 0.407SWC–0.091VPD	338.5	<0.01	0.529**	0.204**	−0.092**	0.113**	−0.129**
Grazed: GPP = 0.246 + 0.0003Rn + 0.004Ta–0.005Ts–0.173SWC–0.027VPD	411.9	<0.01	0.498**	0.072**	−0.112**	−0.072**	−0.04*

^*^indicates that the correlation is significant at the 0.05 level.

^**^indicates that the correlation is significant at the 0.01 level.

## References

[b1] Groisman P. Y. *et al.* The northern Eurasia earth sicence partnership. B. Am. Meteorol. Soc. 90, 671–688 (2009).

[b2] Tan Z., Liu S., Tieszen L. L. & Tachie-Obeng E. Simulated dynamics of carbon stocks driven by changes in land use, management and climate in a tropical moist ecosystem of Ghana. Agric., Ecosyst. Environ. 130, 171–176, 10.1016/j.agee.2009.01.004 (2009).

[b3] Peichl M., Carton O. & Kiely G. Management and climate effects on carbon dioxide and energy exchanges in a maritime grassland. Agric., Ecosyst. Environ. 158, 132–146 (2012).

[b4] Polley H. W., Frank A. B., Sanabria J. & Phillips R. L. Interannual variability in carbon dioxide fluxes and flux-climate relationships on grazed and ungrazed northern mixed-grass prairie. Global Change Biol. 14, 1620–1632, 10.1111/j.1365-2486.2008.01599.x (2008).

[b5] Eaton J. M., McGoff N. M., Byrne K. A., Leahy P. & Kiely G. Land cover change and soil organic carbon stocks in the Republic of Ireland 1851–2000. Clim. Change 91, 317–334 (2008).

[b6] Zeeman M. J. *et al.* Management and climate impacts on net CO_2_ fluxes and carbon budgets of three grasslands along an elevational gradient in Switzerland. Agr. Forest Meteorol. 150, 519–530, 10.1016/j.agrformet.2010.01.011 (2010).

[b7] Schmitt M., Bahn M., Wohlfahrt G., Tappeiner U. & Cernusca A. Land use affects the net ecosystem CO_2_ exchange and its components in mountain grasslands. Biogeosciences 7, 2297–2309 (2010). 23293657 10.5194/bg-7-2297-2010 PMC3535888

[b8] Niu S. *et al.* Seasonal hysteresis of net ecosystem exchange in response to temperature change: patterns and causes. Global Change Biol 17, 3102–3114, 10.1111/j.1365-2486.2011.02459.x (2011).

[b9] Prescher A.-K., Grünwald T. & Bernhofer C. Land use regulates carbon budgets in eastern Germany: From NEE to NBP. Agr. Forest Meteorol 150, 1016–1025, 10.1016/j.agrformet.2010.03.008 (2010).

[b10] Cao G. *et al.* Grazing intensity alters soil respiration in an alpine meadow on the Tibetan plateau. Soil Biol. Biochem 36, 237–243 (2004).

[b11] Chen B. *et al.* The impact of climate change and anthropogenic activities on alpine grassland over the Qinghai-Tibet Plateau. Agr. Forest Meteorol 189, 11–18, 10.1016/j.agrformet.2014.01.002 (2014).

[b12] Owensby C. E., Ham J. M. & Auen L. M. Fluxes of CO_2_ from grazed and ungrazed tallgrass prairie. Rangeland Ecol. Manag. 59, 111–127, 10.2111/05-116r2.1 (2006).

[b13] Bremer D. J., Auen L. M., Ham J. M. & Owensby C. E. Evapotranspiration in a Prairie Ecosystem. Agron. J. 93, 338–348 (2001).

[b14] Shao C., Chen J., Li L. & Zhang L. Ecosystem responses to mowing manipulations in an arid Inner Mongolia steppe: An energy perspective. J. Arid Environ. 82, 1–10 (2012).

[b15] Klein J. A., Harte J. & Zhao X.-Q. Dynamic and complex microclimate responses to warming and grazing manipulations. Global Change Biol. 11, 1440–1451, 10.1111/j.1365-2486.2005.00994.x (2005).

[b16] Enriquez A. S., Chimner R. A. & Cremona M. V. Long-term grazing negatively affects nitrogen dynamics in Northern Patagonian wet meadows. J. Arid Environ. 109, 1–5, 10.1016/j.jaridenv.2014.04.012 (2014).

[b17] McSherry M. E. & Ritchie M. E. Effects of grazing on grassland soil carbon: a global review. Global Change Biol. 19, 1347–1357, 10.1111/gcb.12144 (2013). 23504715

[b18] Zhang G., Zhang Y., Dong J. & Xiao X. Green-up dates in the Tibetan Plateau have continuously advanced from 1982 to 2011. PNAS 110, 4309–4314 (2013). 23440201 10.1073/pnas.1210423110 PMC3600495

[b19] Li C. Q. & Tang M. C. The climate change of Qinghai-Xizang plateau and its neighborhood in the recent 30 years. Plateau Meteorol. 4, 332–341 (1988).

[b20] Marcolla B. *et al.* Climatic controls and ecosystem responses drive the inter-annual variability of the net ecosystem exchange of an alpine meadow. Agr. Forest Meteorol. 151, 1233–1243, 10.1016/j.agrformet.2011.04.015 (2011).

[b21] Shao C., Chen J. & Li L. Grazing alters the biophysical regulation of carbon fluxes in a desert steppe. Environ. Res. Lett. 8, 025012 (2013).

[b22] Kato T. & Tang Y. Spatial variability and major controlling factors of CO_2_sink strength in Asian terrestrial ecosystems: evidence from eddy covariance data. Global Change Biol. 14, 2333–2348, 10.1111/j.1365-2486.2008.01646.x (2008).

[b23] Baldocchi D. *et al.* FLUXNET: A new tool to study the temporal and spatial variability of ecosystem-scale carbon dioxide, water vapor, and energy flux densities. B. Am. Meteorol. Soc. 82, 2415–2434, 10.1175/1520-0477(2001).

[b24] Yuan W. *et al.* Global estimates of evapotranspiration and gross primary production based on MODIS and global meteorology data. Remote Sens. Environ. 114, 1416–1431, 10.1016/j.rse.2010.01.022 (2010).

[b25] Stannard D. I. A theoretically based determination of Bowen-ratio fetch requirements. Bound-Lay Meteorol. 83, 375–406 (1997).

[b26] Wilczak J. M., Oncley S. P. & Stage S. A. Sonic anemometer tilt correction algorithms. Bound-Lay Meteorol. 99, 127–150, 10.1023/a:1018966204465 (2001).

[b27] Falge E. *et al.* Gap filling strategies for defensible annual sums of net ecosystem exchange. Agr. Forest Meteorol. 107, 43–69, 10.1016/s0168-1923(00)00225-2 (2001).

[b28] Webb E. K., Pearman G. I. & Leuning R. Correction of flux measurements for density effects due to heat and water—vapor transfer. Q. J. Roy. Meteor. Soc. 106, 85–100 (1980).

[b29] Reichstein M. *et al.* On the separation of net ecosystem exchange into assimilation and ecosystem respiration: review and improved algorithm. Global Change Biol. 11, 1424–1439 (2005).

[b30] Fu Y. L. *et al.* Depression of net ecosystem CO_2_ exchange in semi-arid Leymus chinensis steppe and alpine shrub. Agr. Forest Meteorol. 137, 234–244, 10.1016/j.agrformet.2006.02.009 (2006).

[b31] Michaelis L. & Menten M. L. Die kinetik der invertinwirkung. Biochem. Z 49, 333–369 (1913).

[b32] Shi P. L. *et al.* Net ecosystem CO_2_ exchange and controlling factors in a steppe - Kobresia meadow on the Tibetan Plateau. Sci. China Ser. D-Earth Sci. 49, 207–218, 10.1007/s11430-006-8207-4 (2006).

[b33] Kato T. *et al.* Carbon dioxide exchange between the atmosphere and an alpine meadow ecosystem on the Qinghai–Tibetan Plateau, China. Agr. Forest Meteorol. 124, 121–134 (2004).

[b34] Frank A. B. & Dugas W. A. Carbon dioxide fluxes over a northern, semiarid, mixed-grass prairie. Agr. Forest Meteorol. 108, 317–326 (2001).

[b35] Frank A. B. Carbon dioxide fluxes over a grazed prairie and seeded pasture in the Northern Great Plains. Environ. Pollut. 116, 397–403, 10.1016/s0269-7491(01)00216-0 (2002). 11822718

[b36] LeCain D. R., Morgan J. A., Schuman G. E., Reeder J. D. & Hart R. H. Carbon exchange and species composition of grazed pastures and exclosures in the shortgrass steppe of Colorado. Agric., Ecosyst. Environ. 93, 421–435 (2002).

[b37] Misson L. *et al.* Functional changes in the control of carbon fluxes after 3 years of increased drought in a Mediterranean evergreen forest? Global Change Biol. 16, 2461–2475 (2010).

[b38] Law B. E. *et al.* Environmental controls over carbon dioxide and water vapor exchange of terrestrial vegetation. Agr. Forest Meteorol. 113, 97–120 (2002).

[b39] Cui X. *et al.* Photosynthetic depression in relation to plant architecture in two alpine herbaceous species. Environ. Exp. Bot. 50, 125–135 (2003).

[b40] Farquhar G. D., Schulze E. D. & Kuppers M. Responses to humidity by stomata of Nicotiana glauca L. and Corylus avellana L. are consistent with the optimization of carbon dioxide uptake with respect to water loss. Funct. Plant Biol. 7, 315–327 (1980).

[b41] Li S. G. *et al.* Net ecosystem carbon dioxide exchange over grazed steppe in central Mongolia. Global Change Biol. 11, 1941–1955 (2005).

[b42] Wang Z., Xiao X. & Yan X. Modeling gross primary production of maize cropland and degraded grassland in northeastern China. Agr. Forest Meteorol. 150, 1160–1167, 10.1016/j.agrformet.2010.04.015 (2010).

[b43] Chen J. *et al.* Biophysical controls of carbon flows in three successional Douglas-fir stands based on eddy-covariance measurements. Tree Physiol 22, 169–177 (2002). 11830413 10.1093/treephys/22.2-3.169

[b44] Lin Y. *et al.* Grazing intensity affected spatial patterns of vegetation and soil fertility in a desert steppe. Agric., Ecosyst. Environ. 138, 282–292 (2010).

[b45] Xu L. K. & Baldocchi D. D. Seasonal variation in carbon dioxide exchange over a Mediterranean annual grassland in California. Agr. Forest Meteorol. 123, 79–96, 10.1016/j.agrformet.2003.10.004 (2004).

[b46] Zhang W. L. *et al.* Biophysical regulations of carbon fluxes of a steppe and a cultivated cropland in semiarid Inner Mongolia. Agr. Forest Meteorol. 146, 216–229, 10.1016/j.agrformet.2007.06.002 (2007).

[b47] Liu J. *et al.* Plants can benefit from herbivory: stimulatory effects of sheep saliva on growth of Leymus chinensis. PLoS One 7, e29259, 10.1371/journal.pone.0029259 (2012). 22235277 PMC3251555

[b48] Craine J. M., Wedin D. A. & Chapin Iii F. S. Predominance of ecophysiological controls on soil CO_2_ flux in a Minnesota grassland. Plant Soil 207, 77–86 (1999).

[b49] Bremer D. J., Ham J. M., Owensby C. E. & Knapp A. K. Responses of soil respiration to clipping and grazing in a tallgrass prairie. J. Environ. Qual. 27, 1539–1548 (1998).

[b50] Reichstein M. *et al.* Climate extremes and the carbon cycle. Nature 500, 287–295, 10.1038/nature12350 (2013). 23955228

